# Frameless Fractionated Stereotactic Radiosurgery for Vestibular Schwannomas: A Single-Institution Experience

**DOI:** 10.3389/fonc.2013.00121

**Published:** 2013-05-17

**Authors:** Sana D. Karam, Alexander Tai, Alexis Strohl, Matthew K. Steehler, Abdul Rashid, Gregory Gagnon, K. William Harter, Ann K. Jay, Sean P. Collins, Jeffrey H. Kim, Walter Jean

**Affiliations:** ^1^Department of Radiation Medicine, MedStar Georgetown University HospitalWashington, DC, USA; ^2^Department of Otolaryngology Head and Neck Surgery, University of RochesterRochester, NY, USA; ^3^Department of Otolaryngology Head and Neck Surgery, MedStar Georgetown University HospitalWashington, DC, USA; ^4^Department of Radiation Oncology, Fredrick Memorial HospitalFredrick, MD, USA; ^5^Department of Radiology, MedStar Georgetown University HospitalWashington, DC, USA; ^6^Department of Neurosurgery, MedStar Georgetown University HospitalWashington, DC, USA

**Keywords:** acoustic neuroma, vestibular schwannoma, cyberknife, fractionated, SRS, radiation

## Abstract

**Objective:** To examine tumor control, hearing preservation, and complication rates after frameless fractionated stereotactic radiosurgery (SRS) in patients with vestibular schwannomas (VS).

**Methods:** Thirty-seven patients treated with fractionated SRS from 2002 to 2011 were retrospectively analyzed. Ninety-five percent were treated with 25 Gy in five fractions, targeting a median tumor volume of 1.03 cc (range 0.14–7.60).

**Results:** With a median follow-up of 4.25 years (range, 15 months–9 years), no tumors required an additional treatment resulting in 100% tumor control rate. Radiographic control rate was 91% in 32 patients at a median follow-up of 3 years. Of the 14 patients with serviceable hearing and with audiograms, the hearing preservation rate was 78% at a median follow-up of 18 months. Twenty-six patients with serviceable hearing pretreatment, were evaluated by a phone survey with a hearing preservation rate of 73% at a 5 year median follow-up. There were two cases that developed both new increased trigeminal parasthesias and facial spasms but there were no cases of facial weakness. Patient had 96% of good to excellent satisfaction rate with the treatment at a median follow-up of 5 years.

**Conclusion:** Frameless fractionated SRS treatment of VS results in good rate of tumor control. Hearing preservation rate and rates of cranial nerve toxicity are comparable to what is reported in the literature. Patients choose this modality because of its non-invasive nature and are generally very satisfied with their long term outcome.

## Introduction

Vestibular schwannomas (VS) are benign tumors arising mostly from vestibular component of the vestibulocochlear nerve. They constitute about 6% of intracranial neoplasms with an incidence of ∼9–13 per million people per year (Murphy and Suh, 2011). Clinically, VS are categorized as sporadic and unilateral, genetic (NF2-associated) and bilateral, or malignant schwannomas. The unilateral or sporadic tumors are by far the most common, making up 95% of VS. VS are characterized by a slow growth pattern with an increase in diameter of 1 mm per year that is most likely to happen during the first 3 years (Moffat et al., 2012). Hearing loss is the most common initial presenting symptoms and is usually followed by tinnitus, disequilibrium, trigeminal nerve dysfunction, vertigo headache, facial nerve dysfunction, and diplopia (Stucken et al., 2012). Symptoms usually arise from tumor progression as it grows through the internal auditory canal through the cerebellopontine angle and eventually leading to compression of neighboring cranial nerves and the brainstem (Stucken et al., 2012). Treatment protocols range from observation to microsurgical resection (MS) or stereotactic radiation therapy (Arthurs et al., 2011). The choice of treatment depends on the likelihood of maximizing local tumor control while preserving hearing function and minimizing cranial nerve toxicity. A recent meta-analysis of comparing long term hearing preservation outcomes has demonstrated superiority of radiation therapy treatment compared with observation (Maniakas and Saliba, 2012). Studies comparing radiotherapy treatments to microsurgery have reported better hearing preservation outcomes of radiosurgery (RS) compared with microsurgery (Pollock et al., 2006) although the debate continues given the heterogeneity of sample and methodology used in the literature. A recent survey of neurotologist reported increase preference of treating these tumors with stereotactic radiotherapy (SRT) over microsurgery (German et al., 2011).

Various radiation therapy techniques using alternative approaches to tumor targeting have been used for the treatment of VS. These include stereotactic radiosurgery (SRS) using Gammaknife or Cyberknife or SRT using a linac-based accelerator. Within these modalities varying radiation doses and fractionation regimens have been studied (Ishihara et al., 2004; Chang et al., 2005; Arthurs et al., 2011; Collen et al., 2011; Hansasuta et al., 2011; Murphy and Suh, 2011; Roos, 2012). Although studies have shown equivalent tumor control rates, improved hearing preservation rates have been demonstrated with lower SRS dose (Yang et al., 2010; Arthurs et al., 2011). The effect of fractionation, however, is less clear. While equivalent tumor control rates have also been noted for single versus multi-fraction delivery sessions, retrospective analyses examining hearing preservation rates have reported mixed results ranging from superior (Andrews et al., 2001) to equivalent hearing preservation rates with dose fractionation (Meijer et al., 2003; Combs et al., 2010). The ability to fractionate the treatment should allow normal tissue, such as the cochlea and brainstem, to recover from radiation leading to better hearing preservation rates. More recently, the Stanford group published their large series update using Cyberknife based robotic fractionated stereotactic radiosurgery (FSRS) with good tumor control, hearing preservation, and non-auditory complication rates (Hansasuta et al., 2011).

In this manuscript we present our long term institutional experience using robotic FSRS but with an alternative previously utilized fractionation regimen (Williams, 2002). Tumor control rates, hearing preservation rates, quality of life, and non-auditory complication rates are reported.

## Materials and Methods

### Patient characteristics

From September 2002 to September 2011, 55 patients with VS were treated with Cyberknife based FSRS at Georgetown University Hospital. All data were reviewed under an institutional review board-approved retrospective protocol. Patients with neurofibromatosis type 2 were excluded from the analysis. A minimum of 12 month follow-up was required to be included in the analysis. Eighteen patients had either no (*n* = 9) or ≥1 year (*n* = 9) follow-up data. Thirty-seven patients with ≥1 year follow-up data were analyzed. Pre and post-treatment radiographic digital imaging was only available on 32 patients. Nineteen patients had pre- and post-audiogram data for analyses, 14 of which had serviceable hearing. Hearing, facial nerve function, tumor volume/mass effect were analyzed with the Gardner and Robertson (1988), House and Brackmann (1985), and Koos et al. (1998) scales, respectively. Twenty-nine patients were reached by phone and perception of hearing preservation as well as overall satisfaction with the treatment was evaluated.

Patient characteristics are summarized in Table 1. The median age was 58 (range 31–85) with a 70% male majority. The laterality was divided almost equally between the left and right side. None of the patients had received any prior treatment for their tumor. Majority of the patients (81%) presented with hearing loss as an initial symptom while ataxia/disequilibrium and tinnitus were presenting symptoms in 57 and 46% of the patients, respectively. None of the patients had any symptoms of facial nerve involvement on presentation.

**Table 1:** **Patient characteristics**.

Age (median, range, years)	58 (31–85)
**GENDER**	***n* (%)**
Male	26 (70)
Female	11 (30)
**LOCATION**	***n* (%)**
Right	18 (49)
Left	19 (51)
**PRIOR SURGERY**	***n* (%)**
Symptoms at presentation	0 (0)
Trigeminal paresthesias	1 (2.7)
Trigeminal neuralgia	0 (0)
House–Brackmann facial nerve function Grade 1	37 (100)
Hemifacial spasms	0 (0)
Hearing loss	30 (81)
Tinnitus	16 (46)
Ataxia/disequilibrium	20 (57)

### Treatment characteristics

Treatment characteristics are presented in Table 2. The median tumor volume was 1.03 cc with a range from 0.14 to 7.60 cc. The vast majority of the patients (95%) were treated with 25 Gy in five sessions while only two patients were treated to 21 Gy in three fractions. The majority of the tumors (54%) were Koos Grade 2. The conformity index (prescribed isodose volume/tumor volume encompassed by the prescription isodose line) and the modified conformity index [(prescribed isodose volume / tumor volume encompassed by the prescription isodose line)/tumor volume] was calculated on all patients (Collins et al., 2006). The median conformality index was 1.60 with a range of 1.01–2.59 while the median prescription isodose line was 80% with a range from 60 to 92%.

**Table 2:** **Treatment characteristics**.

Tumor volume, median (range), cc	1.03 (0.14–7.60)
**KOOS CLASSIFICATION**	***n* (%)**
I	13 (35)
II	20 (54)
III	4 (11)
IV	0 (0)
**SESSION, *n*/TOTAL DOSE, Gy**	***n* (%)**
5/25	35 (95)
3/21	2 (5)

### Radiosurgical technique and follow-up

The CyberKnife FSRS system (Accuray, Inc., Sunnyvale, CA, USA) uses a 6-MV X-band linear accelerator (LA) mounted on a fully articulated robotic arm. During treatment, two orthogonally positioned x-ray detectors provide real-time imaging of bony anatomy allowing for intrafraction movement correction. Treatment was generally administered on an outpatient basis with each treatment lasting ∼45–90 min. Most of the patients received their treatments over the course of five consecutive days.

Patients were immobilized in the supine position with an Aquaplast facemask (WRF/Aquaplast Corp., Wyckoff, NJ, USA). All patients underwent a treatment planning computed tomography (CT) scan (1.25 mm slices) fused with high resolution Fast Imaging Employing Steady State Acquisition (FIESTA) magnetic resonance imaging (MRI) scans. The radiation oncologist, neurosurgeon, or neurotologist, and radiation physicist performed tumor delineation, dose selection, and planning. Inverse planning was used to determine the dose to the target volume while minimizing the dose to normal tissue, especially the cochlea and vestibular organ. Target coverage, coformality index, and dose heterogeneity were examined to evaluate the quality of treatment plans. Informed consent was obtained from all patients.

Patients typically underwent a post-treatment surveillance with an MRI scan, audiogram, and clinic visits 6 months after the completion of FSRS for the first 2 years then annually thereafter. Five years after treatment, follow-up visits were conducted every other year. A follow-up phone interview was conducted for the purposes of this study. Patients were asked the reason for choosing this type of treatment, their recollection of presenting symptomatology, improvement of symptoms, and development of any new symptoms since the treatment. Patients were also asked if their hearing has changed since the treatment and if worsening is noted, whether that has impacted their daily function. Inquiry about any additional treatments and their overall satisfaction with the treatment was also addressed in the phone survey. An attempt was made to call all 37 patients by phone. Twenty-eight patients were surveyed, but only 26 participated in the hearing preservation portion of the survey.

### Statistical analysis

Tumor control rate was assessed using two definitions. Interventional tumor control rate was defined as the absence of the need for additional surgical or radiosurgical intervention according to the Stanford update series (Hansasuta et al., 2011). Radiographic tumor control was defined as progression on the follow-up MRI according to a neuroradiologist interpretation. Kaplan–Meier product-limit method was used to calculate tumor control rate and patients were censored at the time of their last follow-up. Hearing preservation was defined as maintenance of Gardner–Robertson Grade 1– 2 hearing after SRS. Given that the number of events was very small for tumor control correlational analyses was not conducted. For the phone survey, hearing preservation as a perception of same or worse compared to pretreatment baseline. Satisfaction with overall treatment was grouped into four categories: dissatisfied, fine, or good, very good, and excellent. Analyses were performed in SAS version 9.2 (SAS Institute Inc., Cary, NC, USA).

## Results

### Tumor control

**Figure 1:**
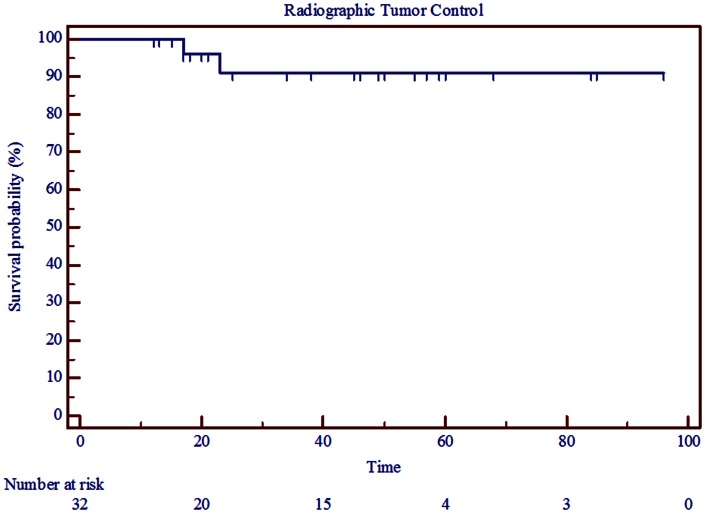
**Kaplan–Meier curve showing radiographic tumor control in 32 patients treated with radiosurgery for vestibular schwannoma between 2002 and 2011**.

The interventional tumor control rate was 100% at a median follow-up of 4.25 years, as none of the tumors displayed enough growth to require additional treatment. The radiographic control rate was 91% at a median follow-up of 3 years and an actuarial follow-up of 5 years (Figure 1; Table 3). The median time to progression was 20 months. Patients who had radiographic progression were Koos Grade 2 or higher, but statistical correlation could not be established due to small number of events.

**Table 3:** **Treatment outcomes**.

Interventional tumor control rate % (*n*, median follow-up)	100% (37, 4.25 years)
Radiographic tumor control rate % (*n*, median follow-up)	91% (32, 3 years)
Crude hearing preservation rate % (*n*, median follow-up)*	78% (14, 18 months)
Koos I	100%
Koos II, III	71.6%
**OTHER COMPLICATIONS**	
Increased trigeminal parasthesias	2 (5)
New facial paresis	0 (0)
Hemifacial spasm	2 (5)
Hydrocephalus	0 (0)
New tinnitus	3 (8)
New ataxia/disequilibrium	1 (2)

*For those with serviceable hearing (GR scale 1–2).

### Hearing preservation

Of the 37 patients, 19 patients had both pretreatment and follow-up audiograms. Five (26%) patients had non-serviceable hearing (GR Grade 3, 4, or 5) before SRS. With a median follow-up of 18 months, 11 of 14 patients maintained serviceable hearing resulting in a crude hearing preservation rate of 78% (Table 3). Further examination revealed that for Koos Grade 1, hearing preservation was 100% while those with Koos Grade 2 and above had a 72% hearing preservation rate. Personal impression of hearing preservation was also assessed by phone on 26 patients. With median follow-up of 5 years, the crude hearing preservation rate was 73% (19 patients reporting same hearing and 6 reporting worsening hearing loss). Twelve patients had missing audiograms (pretreatment, post-treatment, or both). Out of these 12, 10 patients reported no change in hearing while two reported worsening hearing. There were 10 patients with documented serviceable hearing pretreatment (Table 3). Four patients reported worsening hearing despite the fact that the audiogram documentation showed only two patients progressing to the non-serviceable category. Among those with non-serviceable pretreatment audiograms (GR 3–5), three out of four patients reported same hearing whereas one reported worsening hearing (Table 4). When asked how the change in hearing has impacted daily function, 21 patients indicated no impact (81%) while 5 patients indicated worsening daily functioning as a result of their hearing impairment (19%).

**Table 4:** **Patients’ self report of hearing preservation as assessed by a phone survey and correlated with pre- and post-treatment audiograms when available**.

Self report for change in hearing since SRS treatment	No audiogram available	Same (non-serviceable. Pre- and post-treatment GR ≥ 3, and GR maintained)	Same (serviceable, pre and post GR ≤ 2, and GR maintained)	Worse (non-serviceable. Pre and post GR ≥ 3, but GR increased)	Worse (serviceable pretreatment, non-serviceable post-treatment)	
**ASSESSMENT BY AUDIOGRAM (PRE- AND POST-TREATMENT GR CATEGORY)**						
Same	10	3	6	0	0	19 (73.1%)
Worse	2	0	2	1	2	7 (26.9%)
	12 (46.2%)	3 (11.5%)	8 (30.8%)	1 (3.8%)	2 (7.7%)	26

### Non-auditory complications and overall satisfaction

Non-auditory complications are summarized in Table 3. There were two trigeminal nerve complications (5%). Both patients developed trigeminal parasthesias and hemifacial spasms, the latter required treatment with pharmacological agents with resolution of symptoms. There were no reports of trigeminal neuralgia or facial weakness. There were no cases of hydrocephalus. Three patients reported new onset tinnitus, while one with pretreatment tinnitus reported resolution of symptoms. Additionally, seven patients who presented with ataxia/disequilibrium reported resolution of symptom. One patient, however, reported new onset disequilibrium resulting in impairment of her daily functioning.

When asked why they chose this treatment, the vast majority of the respondents cited the non-invasive nature of the procedure and fear of nerve damage during surgery 24/26. Two patients were also inoperable and two cited hearing preservation as the main reason for choosing SRS. The majority of those surveyed were satisfied with the treatment. Thirteen patients responded good or fine (46%), 2 patients responded very good (7%), and 12 patients responded excellent, outstanding, or great (43%). Only one patient who had developed facial spasms was dissatisfied with the treatment (4%).

## Discussion

In this paper we presented our institutional experience with frameless robotic Cyberknife based FSRS. Our results show good tumor control with 100% interventional tumor control rate and 91% radiographic tumor control rate at a median follow-up of 4.25 years. Multiple studies report similar high rates of tumor control with various radiation treatment modalities including Gamma Knife based SRS, LA-based SRS, conventionally fractionated stereotactic radiation therapy, proton beam radiation therapy [reviewed in (Arthurs et al., 2011; Murphy and Suh, 2011)]. Results from publications with the frameless Cyberknife based FSRS using the Cyberknife system have also been similar. Ishihara et al., 2004, reported a 94% radiographic tumor control rate at a median follow-up of 27 months using the same system (Ishihara et al., 2004) while the Stanford series reported a radiographic control rate of 98% at a mean follow-up of 48 months (Chang et al., 2005). In their recent update, the Stanford group reported an interventional tumor control rate of 99 and 96% at 3 and 5 years, respectively with the use of multisession SRS results (Hansasuta et al., 2011). While the latter study represents the largest and most comprehensive experience with this system, a direct comparison of our results and the above studies remains difficult as the adherence to the 2003 Consensus Reporting Standards (Kanzaki et al., 2003) is highly variable between the studies. A recent meta-analysis attempting to examine outcome differences between observation and stereotactic radiation therapy for the management of VS, could not include fractionated SRT as none met the criteria of the meta-analysis (Maniakas and Saliba, 2012).

Our hearing preservation rates are also comparable to what has been published with SRS (Delbrouck et al., 2011; Murphy and Suh, 2011; Roos, 2012) and with frameless Cyberknife FSRS (Ishihara et al., 2004; Chang et al., 2005; Hansasuta et al., 2011). Here we report a hearing preservation rate of 78% at median follow-up of 18 months and 73% based on patient’s report at a median follow-up of 5 years. Not surprisingly we found that tumor control rate for the smaller sized tumors (Koos I) was 100% and is lower for larger tumors. This is similar to the results by Hansasuta et al. (2011), who reported a hearing preservation rate of 85 and 75% at a median follow-up of 3 years for Koos I, and Koos Grade 2 or higher, respectively. Although previous studies with the Cyberknife FSRS system also used a fractionated regimen, the majority of our patients received a different fractionation scheme of 5 Gy delivered in five fractions. The value of fractionated SRS is that it allows for delivery of highly conformal treatment of targets that are in close proximity to critical structures such as the cranial nerves. We hypothesized that further fractionation would improve the therapeutic ratio, thereby reducing the risk of late complications potentially associated with a large single dose or fewer and larger fractions. Indeed, a recent dosimetric comparison of LA-based (BrainLAB) and robotic RS (CyberKnife) systems for VS showed while there is no significant differences in conformity index between the two systems, organs at risk including the choclea and the mesial bone received significantly lower doses with the robotic Cyberknife system (Dutta et al., 2012). This analysis, however, was a dosimetric one and not based on clinical outcomes. Our results show a 5% facial and trigeminal nerve toxicity at a median follow-up of 4.25 years. Rates of new onset permanent and transient facial weakness of 0–5 and 1–10%, respectively, have been reported after single fraction SRS (Delbrouck et al., 2011; Murphy and Suh, 2011). In the Stanford update, Hansasuta et al. (2011) reported at 3.6 year median follow-up a 0.5 and 1.6% trigeminal and facial nerve toxicity rates, respectively, using a fractionation of scheme of 6 Gy delivered in three fractions (Hansasuta et al., 2011). Given the retrospective nature of these studies a conclusion about the superiority of one fractionation regimen over the other cannot be made due to the inherent biases associated with the study design but calls for the need for prospective trials comparing the two fractionation regimens.

Our phone survey demonstrated good to excellent satisfaction rate of 96% at a median follow-up of 5 years. This suggests that the nature and degree of the side effects do not interfere with their quality of life. However, a direct quality of life assessment was not conducted in this study and this question would require further assessment. The discordance between hearing preservation results by self report and by audiogram analysis also highlights this point. Whether this discrepancy is due to the longer median follow time with the self report data than that of audiogram, or whether it is due to a perception of a decline in quality of life remains to be determined. A systemic review of quality of life management of VS comparing MS with radiation treatments showed that although the efficacy of the two seemed equivalent, such significant heterogeneity among the trials existed to a degree that a meta-analysis could not be performed (Gauden et al., 2011). A VS specific quality of life assessment scale developed at the University of Pennsylvania (PANQOL scale) has been validated and shown superiority to the Short Form-36 (SF-36) Health Survey (Shaffer et al., 2010). This will be a potentially a very critical outcome measure in trials examining the correlation between clinical indicators and quality of life outcomes in patients with VS.

## Conclusion

This study has demonstrated the feasibility of using frameless fractionated SRS with 25 Gy in five fractions with good long term tumor control rates. Our results also show this to be an attractive modality for patients with this disease given its non-invasive nature. Hearing preservation rate and rates of cranial nerve toxicity are comparable to what is reported in the literature. Patients were very stratified with their outcome long term. Our retrospective review is, however, limited by potential selection bias, sample size, and heterogeneous patient population. Additionally, the fact that the majority of the tumors in our study were of Koos classification I and II, a group with historically excellent outcomes, biases our results and raises questions about whether an actual benefit for hypofractionation exists for these classifications. Further well-designed, multi-institutional randomized prospective research is necessary to understand this condition, evaluate SRS treatment modalities and fractionation regimens, as well as treatment effect on quality of life.

## Conflict of Interest Statement

Dr. Sean Collins is a clinical consultant for Accuray and Dr. Gagnon is on the clinical advisory board for U.S. Radiosurgery. Actual or potential conflicts of interest do not exist for any other authors.
